# In This Issue

**Published:** 2004

## SOCIAL AND PSYCHOLOGICAL INFLUENCES ON EMERGING ADULT DRINKING BEHAVIOR

Young adulthood is a time of important transitions. During this period, people are forming adult identities, building mature relationships, and preparing for their careers. Increased alcohol use during this time of emerging adulthood may be attributed to a variety of factors, but drinking can undermine the ability of some people to make the developmental transitions typical of this age. Drs. Helene Raskin White and Kristina Jackson examine the developmental changes and related drinking patterns and problems that occur during emerging adulthood as well as the sociodemographic and psychosocial factors that influence drinking in this population. These factors include gender, race/ethnicity, marital status, college, employment, peer and family influences, individual temperament, and attitudes toward drinking. The authors also discuss the need for prevention programs targeted to college students and to young adults not in college, who may be at particularly high risk for alcohol-related problems. (pp. 182–190)

## ALCOHOL USE AND VIOLENCE AMONG YOUNG ADULTS

Young adults experience more violence than other age groups, with bars and clubs being the setting of the most severe violence for young men, and young women tending to encounter the most severe violence in the home. Drs. Brian M. Quigley and Kenneth E. Leonard report that in both environments, intoxicated aggression appears to be provoked by personality differences among people and by characteristics of the triggering situations. Permissive atmospheres in bars increase the probability of intoxicated aggression, and the more alcohol consumed, the greater the likelihood of injury. In domestic violence situations, alcohol use by the husband is predictive of severe violence only in marriages already high in conflict. Many important questions about the relationship between alcohol and violence await further study. Does alcohol reduce a person’s attention to information that would inhibit aggression, as some have hypothesized? Does alcohol have pharmacological effects on decisionmaking in conflict situations that could contribute to violent outcomes? How does alcohol affect people with specific personality traits such as anger and impulsiveness? More information on these questions is needed so that effective prevention and treatment efforts can be developed. (pp. 191–194)

## TRAJECTORIES OF ALCOHOL USE DURING THE TRANSITION TO ADULTHOOD

As young people make the transition from their late teens to adulthood, their drinking practices may follow a variety of tracks, or trajectories. Some young adults will establish lifelong patterns of alcohol use. Others may engage in a particular pattern of alcohol use in their late teen or young adult years that later changes course. Still others may never engage in drinking or heavy drinking. Generally, people increase the amounts they drink as they progress through their early twenties, and decrease their drinking when they take on adult roles. In this article, Drs. Jennifer L. Maggs and John E. Schulenberg describe both the average (or normative) pattern of alcohol use among young adults and the various subgroups of people whose drinking patterns follow different trajectories. They maintain that by identifying common trajectories of alcohol use during the transition to adulthood, researchers can gain a better understanding of how alcohol use disorders originate and evolve, how different trajectories lead to different outcomes, and how to plan prevention, diagnosis, and intervention. (pp. 195–201)

## MATURING OUT OF PROBLEMATIC ALCOHOL USE

After peaking when young people are about 22 years old, heavy drinking rates among both college students and their nonstudent age-mates decline steadily, probably in response to the new roles and responsibilities that come with adulthood. Dr. Patrick M. O’Malley describes key transitions to adulthood and how they are associated either with increased drinking or with “maturing out” of alcohol use. The first major change, moving out of the parental home, often coincides with increasing rates of heavy drinking among many young people, whether they attend college or not. College students then may be exposed to campus social environments that sometimes encourage excessive drinking. As adult responsibilities—such as employment, marriage, and parenthood—mount, problematic drinking declines, partly because of the limitations these responsibilities place on social activities in general and because of changes in young adults’ attitudes toward drinking. Young alcohol users who would be diagnosed as alcohol dependent may be less affected by the more stabilizing, socializing effects of marriage and parenthood because of personality characteristics that make them less attracted to these choices, or because their drinking behavior is less susceptible to such influences. (pp. 202–204)

## ALCOHOL AND THE ADOLESCENT BRAIN: HUMAN STUDIES

Adolescents and young adults who drink heavily could place themselves at risk for alcohol-related impairment of brain development and brain function. As Dr. Susan F. Tapert, Ms. Lisa Caldwell, and Ms. Christina Burke explain, heavy alcohol use in young people has been found to affect neuropsychological functioning, such as memory, attention, planning, and abstract reasoning, although the observed impairment in most cases was not severe. Young people with alcohol use disorders may have subtle abnormalities in the structure of various brain areas, such as the hippocampus, a region that is involved in learning and memory formation. These abnormalities, in turn, may be associated with some loss in brain functioning. A person’s risk for impaired brain development and function is influenced by a number of factors, including genetics and other influences such as drinking patterns and use of other drugs. (pp. 205–212)

## ALCOHOL’S EFFECTS ON THE ADOLESCENT BRAIN: WHAT CAN BE LEARNED FROM ANIMAL MODELS

Research into the effects of alcohol on the developing human brain typically has depended on a variety of animal models—including rats, mice, and other laboratory animals—because both ethical and practical considerations make it impossible to carry out these investigations in humans. Analyses of animal models have demonstrated that adolescents may be more susceptible than adults to some of alcohol’s effects on the brain, especially to alcohol’s memory-impairing effects. As Drs. Susanne Hiller-Sturmhöfel and H. Scott Swartzwelder explain, this enhanced susceptibility may be linked in particular with the hippocampus, an area of the brain that appears to be especially sensitive to alcohol-induced damage. Alcohol interferes with hippocampal function to a greater extent in adolescents than in adults. Conversely, adolescents appear to be less sensitive than adults to some of alcohol’s other effects, such as alcohol-related impairment of motor coordination, alcohol-induced sedation, and susceptibility to withdrawal seizures. (pp. 213–221)

## GENE–ENVIRONMENT INTERPLAY IN ADOLESCENT DRINKING BEHAVIOR

The relative contributions of genetic and environmental factors to adolescents’ drinking behavior are illuminated by two longitudinal studies of Finnish twins reviewed in this article by Drs. Richard J. Rose and Danielle M. Dick. The authors posit that a person’s drinking behavior is a manifestation of an unfolding developmental process involving both genetic and environmental factors, a process that is continuously modulated by gene–environment correlations and interactions. For example, initiation of drinking is determined primarily by environmental factors (e.g., familial environment and peers). Conversely, the establishment of drinking patterns once drinking has been initiated is increasingly influenced by genetic predisposition, and researchers are working to identify specific genes involved. Environmental factors such as place of residence or marital status continue to modify the effects of these genetic factors. (pp. 222–229)

## ENVIRONMENTAL INFLUENCES ON YOUNG ADULT DRINKING

Environmental alcohol control policies may be effective in reducing alcohol use and related problems among young adults, although the population of 18- to 25-year-olds is seldom specifically targeted by these policies or by research evaluating them. According to Drs. Alexander C. Wagenaar and Traci Toomey and Ms. Kathleen M. Lenk, many studies have shown that establishing a minimum legal drinking age (MLDA) of 21 is effective in reducing alcohol consumption and traffic crashes among 18- to 20-year-olds. Underage youth appear to be especially susceptible to prevention measures that limit alcohol availability, specifically price increases. And because young adults frequent retail alcohol establishments more often than members of other age groups, they also may be disproportionately affected by policies that target these outlets, such as limiting the number of retail alcohol establishments in a geographic area, training staff and management in responsible beverage service, and restricting the marketing of alcoholic beverages through special promotions and advertising. The authors describe three comprehensive programs that have used combinations of these measures and list policy recommendations that are supported by current evidence. (pp. 230–235)

## DRINKING AMONG YOUNG ADULTS: SCREENING, BRIEF INTERVENTION, AND OUTCOME

Twenty-five percent of young adult males and 14 percent of females have at some time met the diagnostic criteria for alcohol dependence. Although many young people mature out of problem drinking during early adulthood, excessive drinking during adolescence and young adulthood can have serious and long-lasting negative consequences. Because young people do not tend to identify themselves as having alcohol problems, Drs. Peter M. Monti, Tracy O’Leary Tevyaw, and Brian Borsari suggest that people in this group may be better identified using proactive screening in locations where they are likely to seek treatment related to alcohol problems, such as hospital emergency rooms, college campuses, or workplaces. Young adults engaging in risky levels of alcohol use may respond better to brief, intensive interventions, known as brief motivational interventions, than to traditional, long-term treatments, which originally were designed for adults with more extensive histories of alcohol use. (pp. 236–244)

## INTERNATIONAL PERSPECTIVES ON ADOLESCENT AND YOUNG ADULT DRINKING

International comparisons of both adult and adolescent drinking behaviors analyzed under the auspices of the World Health Organization have demonstrated great differences in alcohol-related variables—such as average alcohol consumption, proportion of drinkers, and drinking patterns—among people in various regions of the world. The European School Survey Project on Alcohol and Other Drugs (ESPAD) has revealed a wide range of behaviors among European adolescents and young adults in terms of age at onset of drinking, prevalence of abstinence, drinking to intoxication, and frequency and amount of drinking. As Dr. Salme K. Ahlström and Ms. Esa L. Österberg report, differences in social norms, the context of drinking occasions, and alcohol pricing policies contribute to the observed variations in drinking patterns. Particularly for the age group of young adults, however, substantial gaps remain in the understanding of drinking behavior. (pp. 258–268)

## COMMUNITY PREVENTION OF YOUNG ADULT DRINKING AND ASSOCIATED PROBLEMS

Whether they are working, attending college, or in the military, young adults are typically part of a community. In this article, Dr. Harold D. Holder examines three research-based community prevention programs that use a combination of environmental strategies to reduce heavy drinking and related problems. Trials of programs provide strong evidence that comprehensive strategies can effect substantial changes in alcohol-related behavior. Research indicates that local policies to reduce young adult drinking or alcohol-related problems are most likely to be effective when they are adequately enforced and when the intended targets of the intervention are aware of both the policies and their enforcement. By restructuring the total alcohol environment in a way that can be self-sustaining, these approaches are likely to be more effective than one-time interventions. (pp. 245–249)

## PREVENTING ALCOHOL-RELATED PROBLEMS ON COLLEGE CAMPUSES

News headlines of alcohol-related injuries and deaths among college students highlight the need for effective prevention measures on college campuses. The National Institute on Alcohol Abuse and Alcoholism Task Force on College Drinking addressed this problem. The Task Force reviewed existing interventions, most of which target individual drinkers rather than the college population as a whole, and issued recommendations on which interventions might be effective in a college setting. Dr. Robert Saltz summarizes these recommendations, which classify potential interventions into four tiers. Of these, only Tier 1 strategies (e.g., cognitive-behavioral skills training, motivational enhancement interventions, or interventions to challenge alcohol expectancies) have been demonstrated effective among college students. Tier 2 strategies, which include enforcement of laws to prevent alcohol-impaired driving, restrictions on alcohol retail outlet density, and responsible beverage service policies, have been successful with other populations and show promise for the college environment. (pp. 249–251)

## ALCOHOL USE AND PREVENTING ALCOHOL-RELATED PROBLEMS AMONG YOUNG ADULTS IN THE MILITARY

Heavy alcohol use remains a persistent problem in the U.S. military and is especially prevalent among young adult (18- to 25-year-old) service members. According to Drs. Genevieve Ames and Carol Cunradi, certain characteristics of the military culture may contribute to heavy drinking in this population. The authors compare the rates of alcohol use among young adults in all four branches of the military with rates for young adult civilians, including college students. In addition to describing the relevant risk factors, such as a workplace culture in which alcohol is accepted as a way to deal with stress, boredom, and loneliness, the article also examines strategies that may help mitigate risk and reduce heavy drinking in this group. Research is needed to evaluate these strategies, which included alcohol use policies, making alcohol use less glamorous, and promoting overall good health. (pp. 252–257)

## ALCOHOL CONSUMPTION AMONG YOUNG ADULTS AGES 18–24 IN THE UNITED STATES: RESULTS FROM THE 2001–2002 NESARC SURVEY

The high prevalence of drinking in young adults is a serious public health concern. Alcohol use among young adults often is associated with a wide variety of risky behaviors and negative consequences, many of which are immediate and tragic. The 2001–2002 National Epidemiologic Survey on Alcohol and Related Conditions (NESARC) presents a unique opportunity to examine young adult drinking for three reasons—the excellent response rate, the oversampling of young adults ages 18–24, and the inclusion of college-related group housing. Mr. Chiung M. Chen and Drs. Mary C. Dufour and Hsiao-ye Yi provide a broad overview of the nature of young adult drinking in the United States using data from the NESARC survey. According to these data, in 2001–2002, over three-quarters of young adults ages 21–24 were current drinkers, as were nearly two-thirds of those ages 18–20, despite the fact that the legal drinking age is 21. More than half of young adult men exceeded the recommended daily drinking limit, as did two-fifths of the young adult women. Drinking that exceeds this daily limit is likely to impair both mental and physical performance. Over the past decade there has been an increase in the number of young people drinking 5 or more drinks on at least 12 occasions during the past year—which helps to explain the increased risk of injury and other acute negative consequences so common today among college students ages 18–24. (pp. 269–280)

